# Left Atrial Ejection Fraction Assessed by Prior Cardiac CT Predicts Recurrence of Atrial Fibrillation after Pulmonary Vein Isolation

**DOI:** 10.3390/jcm10040752

**Published:** 2021-02-13

**Authors:** Reinhard Kaufmann, Richard Rezar, Bernhard Strohmer, Bernhard Wernly, Michael Lichtenauer, Wolfgang Hitzl, Matthias Meissnitzer, Klaus Hergan, Marcel Granitz

**Affiliations:** 1 Department of Radiology, University Hospital Salzburg, Paracelsus Medical University of Salzburg, 5020 Salzburg, Austria; m.meissnitzer@salk.at (M.M.); k.hergan@salk.at (K.H.); m.granitz@gmx.at (M.G.); 2 Clinic of Internal Medicine II, Department of Cardiology, Paracelsus Medical University of Salzburg, 5020 Salzburg, Austria; r.rezar@salk.at (R.R.); b.strohmer@salk.at (B.S.); b.wernly@salk.at (B.W.); m.lichtenauer@salk.at (M.L.); 3 Research Office (Biostatistics), Paracelsus Medical University of Salzburg, 5020 Salzburg, Austria; wolfgang.hitzl@pmu.ac.at

**Keywords:** left atrial transport function, atrial fibrillation, pulmonary vein isolation, outcome, prediction, cardiac computed tomography

## Abstract

Assuming that atrial fibrillation (AF) is associated with left atrial remodeling and dysfunction, we hypothesize that left atrial and left atrial appendage ejection fractions (LAEF and LAAEF) are useful and may be more sensitive outcome predictors of pulmonary vein isolation (PVI). Fifty patients who underwent PVI at our institution with available pre-interventional cardiac computed tomography (CT) for procedure planning were included in this retrospective study. The patients were separated into two groups by recurrence and non-recurrence of AF and subgroups of paroxysmal and persistent AF. Semiautomatic volumetric analysis of the left atrium was used to calculate morphological and functional parameters and optimal cut-offs were calculated using the Youden index. LAEF (accuracy 94%, sensitivity 67%) and LAAEF (accuracy 90%, sensitivity 67%) were significantly reduced in patients with AF recurrence (16% vs. 36%, *p* = 0.00002; 16% vs. 42%, *p* = 0.000002), and in the subgroup analysis, the functional parameters were independent from AF type (paroxysmal and persistent). With a cut-off of <23% for both LAEF and LAAEF (area under the curve (AUC) 0.94, 95%CI 0.84–0.99 and AUC 0.96, 95%CI 0.86–0.99, respectively), AF recurrence occurred in 77.8%, within a mean follow-up period of 229 days. In conclusion, left atrial function on prior cardiac CT offers useful parameters for predicting AF recurrence after PVI.

## 1. Introduction

Pulmonary vein isolation (PVI) is a well-established interventional procedure in the treatment of atrial fibrillation (AF). With recurrence rates of 20–50% [1] and a complication rate of around 10.5% [2], prediction of the procedural outcome is of special interest.

Hemodynamic studies have shown that 25–30% of left ventricular (LV) stroke volume (SV) is contributed by the left atrium (LA), and in numerous cardiovascular diseases, LA dilatation occurs at a certain stage [3]. In the beginning, LA dilatation increases SV; however, after reaching a critical volume, the transport function is reduced due to an unfavorable Frank–Starling relationship [4], and in advanced stages, the conduit function takes over [3]. It is common knowledge that higher LA volumes (LAVs) favor the occurrence of AF due to cardiac remodeling, the emergence of ectopic electrical foci and, in the end, electrical heterogeneity due to fibrosis. Furthermore, a recent meta-analysis showed that high LAV increases the likelihood for AF recurrence after PVI [5].

A new and objective approach for measuring LA function is LA strain imaging, though good image quality and high frame rates are paramount [6]. A good correlation of higher atrial strain rates and maintenance of sinus rhythm after electrical external cardioversion, as well as after PVI, was reported [7,8,9]. Magnetic resonance imaging (MRI) is another radiation-free method which allows differentiated imaging of soft tissue and was already described as a good method for predicting successful PVI by assessing late gadolinium enhancement (LGE), possibly associated with low-voltage tissue and scarring [10]. Major restrictions for its use are limited reproducibility of atrial LGE imaging [11,12], higher costs and medical contraindications (e.g., metal implants).

Cardiac computed tomography (CT) for AF ablation assessment has the advantage of additional evaluation of other cardiovascular conditions, such as coronary artery disease [13,14]. This might have a direct impact on the management of patients with atrial fibrillation because the coexistence of AF and coronary artery disease worsens the prognosis [15], whereas coronary revascularization may reduce the recurrence of AF [16]. Aside from that, advances in technology and optimization of CT protocols in recent years contributed to significantly lower radiation dose and contrast volume, without compromising image quality [17].

Impaired LA transport function after catheter ablation was already described in patients with chronic AF, using cardiac CT [18]. Another study discovered that decreased LA function is independently associated with presence of AF [19]. Wolf et al. were the first to evaluate left atrial function with cardiac CT based on an automatic approach using 3D volume segmentation [20]. Yet the prediction of the outcome of PVI with preprocedural cardiac CT was not investigated in previous studies.

We hypothesize that left atrial and left atrial appendage ejection fraction (LAEF and LAAEF) are additional and maybe even more sensitive parameters in predicting the outcome of PVI than left atrial volume (LAVmax).

## 2. Materials and Methods

### 2.1. Demographic and Clinical Data

All patients with persistent or paroxysmal AF who underwent PVI with either cryoablation or radiofrequency catheter ablation and with retrospectively gated cardiac CT for procedure planning between January 2015 and December 2018 were included in this retrospective study. A positive ethics committee vote in accordance with the Helsinki Declaration was obtained (No. 415-E/2437/8-2019, Ethics Commission Salzburg; chairperson: Alexander Hönel). Baseline characteristics and clinical information were collected for each patient. The latest available follow-up examinations with 24-h Holter ECG were used to screen for recurrence of AF. The success of pulmonary vein isolation was defined as clinical freedom of symptomatic AF together with sinus rhythm on the available Holter ECG recordings during follow-up examinations.

### 2.2. Imaging Data

Every patient routinely received a pre-interventional cardiac CT for procedural planning and as a dataset for intraoperative navigation in multiple cardiac phases (Somatom Definition AS+, Siemens, Erlangen, Germany). No medication was administered to reduce the heart rate prior to the examination. A bolus-tracking technique was used with a 100-mL bolus of non-ionic iodinated contrast media followed by 70 mL saline solution injected at a flow rate of 3.5–5 mL/sec. Data acquisition ranged from the tracheal bifurcation to the diaphragm. Retrospective ECG triggering was used for these patients with this CT machine because atrial fibrillation was generally expected and, therefore, prospective ECG gating was considered problematic. Thus, the scan spanning was the entire cardiac cycle from 0% to 90% in steps of 10%.

**Figure 1:**
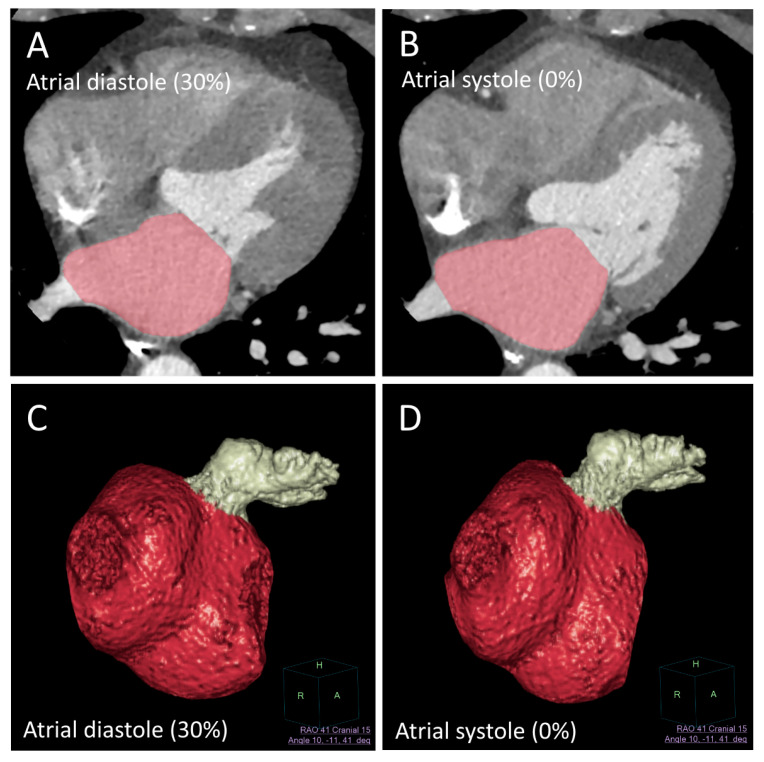
Automatic left atrial segmentation (red) as proposed by Wolf et al. [20] (electrophysiology/EP planning tool, Intellispace Portal, Philips, Best, The Netherlands) with additional manual segmentation of the left atrial appendage (LAA) (white). (**A**,**B**) demonstrate the automatic segmentation of the left atrium (LA) in atrial systole and diastole. (**C**,**D**) demonstrate the 3D surface rendering of the LA and LAA in systole and diastole.

The automatic 3D volume segmentation method of Wolf et al. [20] was used to perform the measurements in atrial diastole (LAVmax, LAAVmax) and atrial systole (LAVmin, LAAVmin), defined by the radiologist’s visual interpretation concerning maximum and minimum volumes of the left atrium (LAVmax, LAVmin), demonstrated in Figure 1. An image post-processing, electrophysiology planning tool was used for segmentation (EP-planning, Intellispace Portal, Philips, Best, The Netherlands). Additionally, left atrial appendage maximum and minimum volumes were measured separately (LAAVmax, LAAVmin) as well as the indexed values in conjunction with body surface area (BSA). All measurements were performed by two independent radiologists, a cardiac radiologist with 10 years of experience and a radiology resident with one year experience in cardiac imaging. Both readers were blinded for clinical outcome, which was separately evaluated by a cardiologist. The functional parameters, namely left atrial stroke volume (LASV; ml; left atrial appendage excluded), left atrial appendage stroke volume (LAASV; ml), left atrial ejection fraction (LAEF; %; left atrial appendage excluded) and left atrial appendage ejection fraction (LAAEF; %), were calculated based on these measurements.

### 2.3. Statistical Analysis

Data were checked for consistency and continuous distributed variables were tested for normality. Based on the latest available post-interventional Holter ECG, the patients were retrospectively divided into two groups: Group A with sinus rhythm and group B without sinus rhythm (= recurrence of AF), as well as subgroups based on AF type (paroxysmal or persistent AF). Differences between the groups were analyzed using independent t-tests with and without the assumption of variance homogeneity, Fisher’s Exact test and Pearson’s Chi-Squared test. Morphological (LAVmax, LAVImax, LAAVmax and LAAVImax) and functional parameters (LASV, LAEF, LAASV and LAAEF) were analyzed for accuracy, sensitivity, specificity, negative predictive value (NPV) and positive predictive value (PPV) with corresponding 95% CI (Pearson–Clopper). Logistic regression analysis was used to compare LAEF to LAVmax in uni- and multivariate models. Final comparisons of the performances (e.g., sensitivities) were made using NAM’s RMLE tests for paired proportions. To analyze interobserver reliability, mixed models with intraclass correlation coefficients based on Cronbach’s alpha and corresponding tests were applied. Receiver operating characteristic (ROC) analysis was performed and the area under the curve (AUC) with respective 95% CIs was calculated. Optimal cut-offs were calculated using the Youden index. All tests were two-sided. *p* < 0.05 indicated a statistically significant difference.

## 3. Results

### 3.1. Demographics and Clinical Data

In total, 152 patients with symptomatic paroxysmal or persistent AF were treated with PVI at our hospital from 2015 to 2018. Of them, 98 patients did not have preprocedural cardiac CTs with retrospective ECG gating including the entire cardiac cycle (0% to 90%); therefore, they could not be included in this retrospective investigation. Four of the 54 remaining patients had to be excluded because of motion artifacts involving the left atrial walls, probably caused by atrial fibrillation during the CT scan (7.4%). Consequently, 50 male and female patients with a mean age of 57.4 years in group A (non-recurrence) and 60.2 years in group B (AF recurrence) were finally included in this study. Demographic and clinical data for both groups are summarized in Table 1. Follow-up examinations with 24-h Holter ECG were performed at an average of 229 days after ablation (range 74 to 1175 days; SD 236 days). A significant difference was found concerning paroxysmal and persistent AF (85% vs. 33%, *p* = 0.003); thus, a subgroup analysis concerning the type of AF was performed. Further significant differences were found between both groups with respect to heart rate and corrected QT interval (QTc). To analyze both parameters as possible covariates for LAEF (%) and LAAEF (%) when comparing both groups, general linear models were applied. When using heart rate as a covariate, LAEF and LAAEF were still significantly associated with AF recurrence in the group comparisons (*p* < 0.001 each). Furthermore, when using QTc as a possible covariate for LAEF and LAAEF, a significant association with the primary outcome was shown (*p* < 0.001 each).

### 3.2. Cardiac CT Parameters

**Figure 2:**
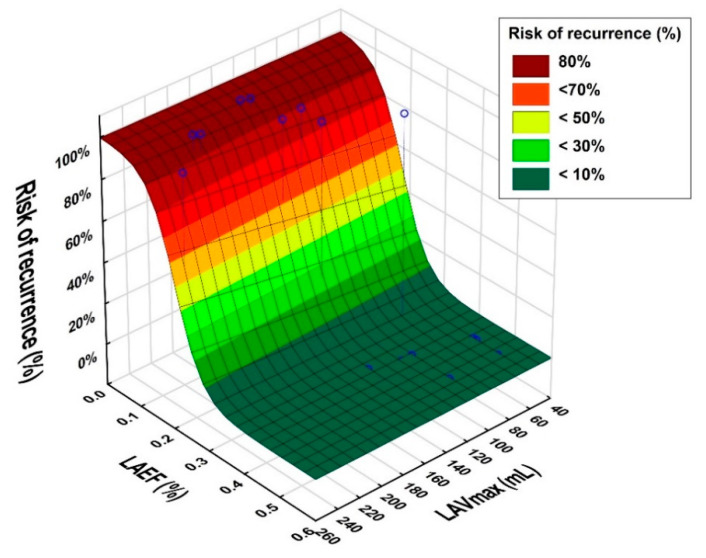
Logistic regression model for risk of recurrence (%) with left atrial ejection fraction (LAEF) (%) to left atrial volume (LAVmax) (mL) as outcome predictors. For example, a patient with an LAEF of 40% and LAVmax of 100 mL had a risk of recurrence of less than 10% (dark green area). However, a patient with lower values of LAEF, e.g., 10%, and the same value of LAVmax of 100 mL had a high risk of recurrence (>80%). The figure also illustrates that LAEF had more predictive power than LAVmax in this study population.

Significant differences were found for all morphological and functional parameters between the non-recurrence group (A) and the recurrence group (B). LAVmax, LAVImax, LAAVmax and LAAVImax were significantly higher in group A, whereas LASV (25.0 mL vs. 43.4 mL, *p* = 0.0008), LAEF (16% vs. 36%, *p* = 0.00002), LAASV (2.4 mL vs. 4.1 mL, *p* = 0.015) and LAAEF (16% vs. 42%, *p* = 0.000002) were significantly lower in group B. The highest accuracy was found for LAEF at 94% and LAAEF at 90%, compared to 86% for LAVmax. Sensitivity for LAEF and LAAEF was also higher compared to LAVmax with 67% vs. 33%, but with no significant difference in this study population (*p* = 0.18; NAM’s RMLE, two-sided). However, in a multivariate model, the combination of LAEF and LAVmax improved the sensitivity with statistical significance to 78% (*p* = 0.046) when compared to LAVmax alone (33%), as illustrated in Figure 2. The performance analysis is demonstrated in Table 2. The AUC for LAEF was 0.94 (95%CI 0.84–0.99) with a cut-off of 23%, and for LAAEF, 0.96 (95%CI 0.86–0.99), equally with a cut-off of 23%. When LAEF and LAAEF were below 23%, AF recurrence was predicted in seven out of nine cases (78.8%). When LAEF and LAAEF were equal to or above 23%, non-recurrence would have been successfully predicted in 36 out of 41 cases (87.8%; mean follow-up period of 229 days).

### 3.3. Paroxysmal vs. Persistent

**Figure 3:**
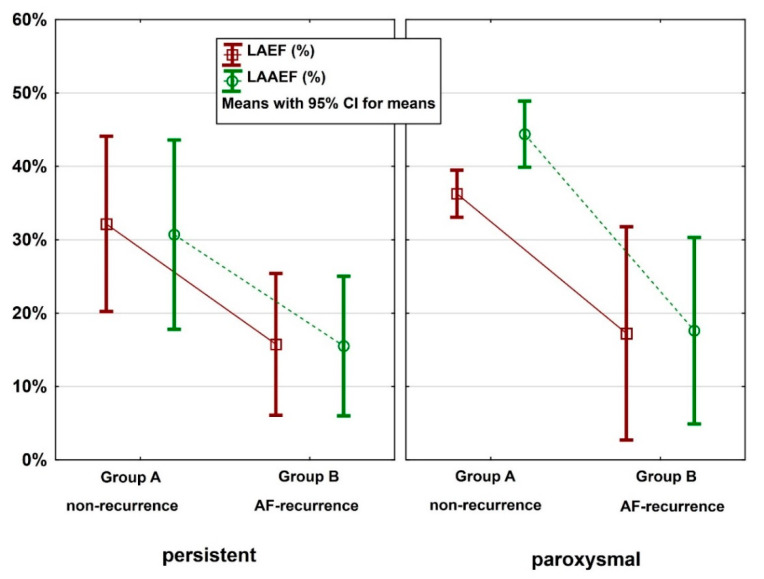
Box-plots of LAEF and left atrial appendage ejection fraction (LAAEF) (%) for non-recurrence (group A) vs. AF recurrence (group B), separated into the subgroups paroxysmal and persistent AF.

Overall, 38 patients were diagnosed with paroxysmal AF and 12 patients with persistent AF. In a subgroup analysis, the functional parameters LAEF and LAAEF were still significantly reduced in the AF recurrence group (B) for both paroxysmal and persistent AF. In the subgroup of paroxysmal AF, LAEF was significantly reduced to 20% (±6%) in group B vs. 40% (±10%) in group A (*p* = 0.002) and LAAEF was reduced to 20% (±5%) in group B vs. 40% (±10%) in group A (*p* = 0.001). In the subgroup with persistent AF, LAEF was significantly reduced to 16% (±9%) in group B vs. 32% (±11%) in group A (*p* = 0.021) and LAAEF was reduced to 16% (±9%) in group B vs. 31% (±12%) in group A (*p* = 0.035). A significant reduction in LASV was also observed in patients with paroxysmal AF (25.8 mL ± 4.7 mL vs. 44.2 mL ± 11.1 mL, *p* = 0.008). The only significant morphological parameter that increased was LAVImax in group B in patients with persistent AF (82.8 mL/m² ± 23 mL/m² vs. 57.9 mL/m² ± 5 mL/m², *p* = 0.045). All other morphological parameters, including LAVmax, were not significant in the subgroup analysis. The results are summarized in Table 3 and Figure 3.

### 3.4. Ablation Technique

A total of 152 consecutive patients underwent pulmonary vein isolation (PVI) for interventional treatment of symptomatic atrial fibrillation from 2015 to 2018. Depending on the discretion of the operator, the PVI was performed with the cryoablation technique (28 mm balloon size only, Arctic Front Medtronic, Dublin, Ireland) in 111 cases (73%) and with radiofrequency (RF) energy in 41 cases (27%) using an irrigated catheter with a contact sensor (25–30 W). Wide-area circumferential ablation (WACA) was performed to avoid the PV ostia with the help of a three-dimensional mapping system (Ensite Velocity, Minnesota, USA) and integration of left atrial CT anatomy. The endpoint for both ablation techniques was exit and entrance block of the pulmonary veins after a waiting time of at least 30 min, using pacing maneuvers and a multipolar circumferential mapping catheter. Additional left atrial lines were applied in four cases due to left atrial flutter. In two cases, focal ablation was delivered for the elimination of a right- and left-sided accessory pathway. In addition to the PVI, cavotricuspid isthmus ablation was performed within the same procedure in a total of 41 patients (27%), who presented clinically with typical atrial flutter and atrial fibrillation. For right atrial flutter ablation, RF energy and an irrigated ablation catheter (35–40 W) were used to achieve bidirectional transisthmus conduction block.

### 3.5. Interobserver Reliability

**Figure 4:**
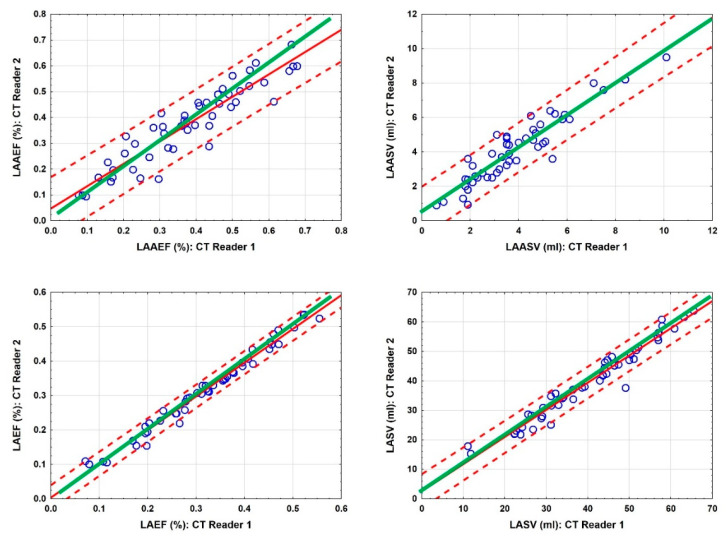
Scatterplots of LAAEF (%), left atrial appendage stroke volume (LAASV) (ml), LAEF (%) and LASV (ml) for CT readers 1 and 2. Green lines indicate the agreement of both readers; red solid lines indicate regression lines with 95% confidence limits (dotted lines).

The correlation between both readers was extremely high for LASV (Cronbach’s alpha 0.988, 95%CI: 0.98–0.99), for LAEF (Cronbach’s alpha 0.995, 95%CI: 0.99–1.0) and for LAAEF (Cronbach’s alpha 0.963, 95%CI: 0.93–0.98). LAASV had significant differences in the mean values (*p* = 0.009), but the correlation between the readers was still very high (Cronbach’s alpha 0.961, 95%CI: 0.92–0.98), illustrated in Figure 4.

## 4. Discussions

A number of previous studies demonstrated that the left atrial transport function constitutes an essential element in the electro-mechanical integrity of the heart. AF, in the long run, causes electrical and mechanical remodeling, leading to left atrial dysfunction. Our data showed that not only left atrial morphology (including left atrial volume) but also left atrial function measured by cardiac CT is significantly altered in patients with recurrence of atrial fibrillation. The present results further demonstrate that even transport function of the left atrial appendage correlates with procedural success of PVI. Sensitivity of LAEF was higher than LAVmax (67% vs. 33%), although not significant in this study population. Nevertheless, the combination of LAEF as a functional parameter and LAVmax a as morphological parameter in a multivariable model significantly improved the sensitivity of AF recurrence prediction up to 78%. Furthermore, and most interestingly, the subgroup analysis for paroxysmal and persistent AF revealed that the functional parameters (LAEF, LAAEF) are still significant concerning AF recurrence, whereas the morphological parameters are not. An optimal cut-off for both LAEF and LAAEF was calculated at 23%, which predicted AF recurrence of 78.8% within a mean follow-up period of 229 days. In other words, only 22.2% of the patients with reduced transport function were free of AF recurrence during the follow-up period. Predicting the outcome of pulmonary vein isolation with more sensitive parameters might have clinical implications for therapy planning in patients with atrial fibrillation, because the risks of an invasive procedure have to be weighed against the potential procedural outcome. Based on these results, left atrial transport function offers such additional parameters that might be beneficial for PVI planning in the future.

The measurements with cardiac CT showed an excellent interobserver reliability. Significant differences were found only for LAASV concerning the mean values, suggesting that the absolute measurements of the left atrial appendage (mL) were more dependent on the CT reader’s interpretation, whereas the relative measurements (%) were less so. This was probably caused by the interpreter-based definition of the LAA ostium. However, the correlation was still excellent for LAASV and LAAEF (Cronbach’s alpha 0.961 and 0.963) as well as for LASV and LAEF (Cronbach’s alpha 0.988 and 0.995). Besides the CT-based analysis, functional parameters of the left atrium may also be assessed with MRI and echocardiography. Therefore, further studies on this topic could evaluate left atrial transport function in a multimodality imaging concept.

CT imaging in patients with atrial fibrillation is often considered problematic, but recent studies have shown high image quality, low radiation exposure and high diagnostic accuracy for coronary CT in patients with chronic AF, similar to the patients with sinus rhythm [21,22]. In our study, only four of 54 patients (7.4%) had to be excluded because of motion artifacts involving the left atrial walls, probably caused by atrial fibrillation during the examination. Although this might be a potential limitation of CT imaging, it applies to MRI as well. In addition, incidental CT findings such as LA or LAA thrombosis might influence the therapy regimen, as non-vitamin K antagonist oral anticoagulants (NOACs) are now considered as a first-line therapy in AF patients with documented LA or LAA thrombus [23]. A critical point, however, is the CT protocol. Retrospective ECG gating was used in these patients because atrial fibrillation was generally expected, and therefore, prospective ECG gating was considered problematic. Meanwhile, the CT machine used in this study was replaced and prospective ECG gating was routinely established to reduce the radiation dose. However, this retrospective study is based on non-ECG-gated imaging data.

The main limitation of our study is the relatively low number of included patients, limiting the statistical power of this investigation. Nevertheless, the number of included participants is comparable to other publications in the past. Certainly, a prospective study design with multimodality imaging, standardized follow-up examinations and evaluation of quality of life could have improved quality rating.

Interestingly, heart rate and QTc time also showed statistically significant differences. Whether these findings are coincidental due to the small number of included patients or whether they contributed to rhythm instability and LA function is not clear and has to be investigated in larger study populations in the future. However, in the covariate analysis, LAEF and LAAEF were still significant after adjustments for heart rate and QTc interval.

Cardiac magnetic resonance imaging (CMR) is the only method of non-invasive assessment of atrial fibrosis and LGE is known as a significant predictor of AF recurrence after PVI. However, the reproducibility of atrial LGE imaging seems to be limited and it is not implemented in every center’s clinical routine [11,12]. Cardiac CT for electrophysiology planning, in contrast, offers additional clinical information, for example, on coronary artery disease [13,14], which might have a direct impact on the management of these patients [15,16] at continuously decreasing radiation doses [17]. Nevertheless, it is important to note that evaluation of left atrial transport function is not limited to one certain imaging modality.

## 5. Conclusions

Prior to PVI, LAEF and LAAEF assessed by cardiac CT for procedure planning were significantly reduced in patients with AF recurrence, independently of AF type (paroxysmal or persistent). A cut-off of <23% predicted AF recurrence in 78.8% of patients within a mean follow-up period of 229 days. CT measurements were highly reproducible between two radiologists. Furthermore, there is a trend that LAEF is more sensitive than LAVmax, which suggests that functional parameters should be further investigated in larger study populations in the future.

## Figures and Tables

**Table 1:** Comparison of demographic and clinical baseline characteristics for both groups (group A: sinus rhythm; group B: atrial fibrillation (AF) recurrence).

	**Group A Non-Recurrence**		**Group B AF Recurrence**		***p***
Total number of patients	41		9		
Sex (female)	9 (22%)		4 (44%)		0.19
Radiofrequency vs. cryoablation	5 (12%)		3 (33%)		0.14
Paroxysmal vs. persistent AF	35 (85%)		3 (33%)		0.003 *
Additional ablation	12 (29%)		1 (11%)		0.41
Coronary artery disease	26 (63%)		6 (67%)		0.89
Arterial hypertension	23 (56%)		8 (89%)		0.072
Stroke in history	4 (10%)		1 (11%)		0.88
Diabetes mellitus	37 (93%)		8 (89%)		0.57
Alcohol	10 (24%)		2 (22%)		0.79
Smoking	11 (27%)		2 (22%)		0.90
Antiarrhythmic agents, class Ic	8 (24%)		1 (13%)		0.66
Antiarrhythmic agents, class II	28 (82%)		8 (89%)		0.58
Antiarrhythmic agents, class III	5 (15%)		1 (13%)		1.0
Anticoagulation with VKA	8 (20%)		4 (44%)		0.19
Anticoagulation with NOAC	33 (80%)		5 (56%)		0.19
	**Mean**	**SD**	**Mean**	**SD**	***p***
Age (years)	57.4	10.84	60.2	12.7	0.55
Agatston Score	149.2	242.9	68.5	115.8	0.15
CHA2DS2-VASc Score	1.59	1.09	2.33	1.41	0.19
HAS-BLED Score	0.46	0.70	0.78	1.03	0.20
Hemoglobin (g/dL)	15.11	1.15	15.40	1.63	0.53
Leukocytes (G/L)	6.96	1.83	6.66	1.48	0.64
Creatinine (mg/dL)	0.97	0.22	0.96	0.15	0.89
TSH (mU/L)	2.42	2.07	1.54	0.82	0.24
CK (U/L)	137.31	101.86	149.33	46.98	0.73
pBNP (pg/mL)	393.62	871.06	744.49	392.79	0.31
Hba1c (%)	5.51	0.44	5.44	0.32	0.68
LDL (mg/dL)	113.54	43.92	99.60	50.77	0.41
HR (beats/min)	64.3	17.6	84.1	20.5	0.006 *
QRS (ms)	100.3	14.7	106.5	27.0	0.93
QTc (ms)	423.4	30.2	459.8	30.4	0.003 *
LV ejection fraction (%)	56.1	2.37	52.11	7.61	0.17
Septal thickness (mm)	9.05	1.79	10.1	1.9	0.12
LV end-diastolic diameter (mm)	53.39	5.05	53.44	6.16	0.98

Significant differences marked with *. Anti-arrhythmic agents were given according to the Vaughan Williams classification. Electrocardiographic parameters (heart rate (HR), QRS, QTc) were determined by 12-lead ECG after the pulmonary vein isolation (PVI). Ejection fraction was determined by means of echocardiography, septal thickness and LV end-diastolic diameter by computed tomography (CT). Abbreviations: AF: atrial fibrillation; CK: creatine kinase; HR: heart rate; LDL: low-density lipoprotein; LV: left ventricular; NOAC: new oral anticoagulants; pBNP: pro-brain natriuretic peptide; TSH: thyroid-stimulating hormone; VKA: vitamin K antagonists.

**Table 2:** Measurements and performance analysis of morphological and functional cardiac computed tomography (CT) parameters (group A: non-recurrence, group B: AF recurrence, NPV = negative predictive value, PPV = positive predictive value).

**Morphological Parameters**				
	**LAVmax (mL)**	**LAVImax**	**LAAVmax (mL)**	**LAAVImax**
Mean A	125.8 ± 29.4	60.6 ± 14.0	10.0 ± 3.54	4.96 ± 1.87
Mean B	163.7 ± 49.6	81.4 ± 22.7	14.3 ± 4.4	7.07 ± 1.87
*p*	0.036	0.026	0.003	0.004
Accuracy (%)	86	88	82	84
Sensitivity (%)	33	44	22	33
Specificity (%)	98	98	95	95
NPV (%)	87	89	84	86
PPV (%)	75	80	50	60
**Functional Parameters**				
	**LAEF (%)**	**LASV (mL)**	**LAAEF (%)**	**LAASV (mL)**
Mean A	36 ± 10	43.4 ± 11.48	42 ± 14	4.1 ± 1.91
Mean B	16 ± 8	25.0 ± 11.2	16 ± 8	2.4 ± 1.39
*p*	0.00002	0.0008	0.000002	0.015
Accuracy (%)	94	88	90	86
Sensitivity (%)	67	56	67	22
Specificity (%)	100	95	95	100
NPV (%)	93	90	93	85
PPV (%)	100	71	75	100

**Table 3:** Morphological and functional cardiac CT parameters separated into subgroups of paroxysmal and persistent AF (group A: non-recurrence, group B: AF recurrence). Significant differences marked with *.

**Paroxysmal AF**			
	**Mean Group A**	**Mean Group B**	***p***
LAVmax (mL)	126.1 ± 31	157.5 ± 42.2	0.11
LAVImax (mL/m²)	61.1 ± 14.8	78.6 ± 26.7	0.07
LASV (mL)	44.2 ± 11.1	25.8 ± 4.7	0.008 *
LAEF (%)	0.4 ± 0.1	0.2 ± 0.1	0.002 *
LAAVmax (mL)	9.8 ± 3.5	13.7 ± 1.9	0.06
LAAVImax (mL/m²)	4.8 ± 1.8	6.8 ± 1.5	0.08
LAASV (mL)	4.2 ± 1.9	2.4 ± 0.9	0.11
LAAEF (%)	0.4 ± 0.1	0.2 ± 0.1	0.001 *
**Persistent AF**			
LAVmax (mL)	126.58 ± 17	166.85 ± 56.5	0.15
LAVImax (mL/m²)	57.93 ± 5	82.83 ± 23	0.045 *
LASV (mL)	40.60 ± 14.6	24.65 ± 13.8	0.08
LAEF (%)	0.32 ± 0.11	0.16 ± 0.1	0.020 *
LAAVmax (mL)	11.45 ± 3.74	14.53 ± 5.4	0.28
LAAVImax (mL/m²)	5.61 ± 2.12	7.20 ± 2.2	0.23
LAASV (mL)	3.60 ± 2.1	2.38 ± 1.7	0.29
LAAEF (%)	0.31 ± 0.1	0.16 ± 0.1	0.035 *

## Data Availability

The data presented in this study are available on request from the corresponding author. The data are not publicly available due to privacy restrictions.
